# Improved Hypodermic Syringe

**Published:** 1891-01

**Authors:** 


### Improved Hypodermic Syringe—Aseptic Syringe.

In the *Medical and Surgical Reporter*, Sept. 28, 1889, Dr. J. J. Thomas, of Youngstown, Ohio, described a new method of administering hypodermic injections, and in a recent number of the *Reporter*, he calls attention again to his method which has met the approval and recommendation of no less an authority than Dr. John B. Roberts, the well-known Philadelphia Surgeon and Professor of Surgery in the Woman's Medical College, who has styled the syringe and suggests that it be named the "Aseptic" injector. We append here the description:

Take the rubber or bulbous portion of an ordinary medicine dropper, using care to select one of some strength and firmness. Insert into its open end the proximate extremity of a hypodermic needle. The fitting is not expected to be close, but can easily be rendered air tight by a few turns of thread, or preferably, a fine rubber band—Faber's No. 10 is strong enough. Now compress the bulb to flatness to expel the air, and dip the needle-point into some water, release the pressure upon the bulb, and it fills with water in the space of twenty-five or thirty seconds. Now by pressure discharge the water into a teaspoon, add your tablet, make the solution and draw it up again into the bulb, and you are ready for injecting. Pinch up a fold of the skin in the ordinary manner, thrust in the needle and deliver the injection by firm pressure upon the bulb, holding it transeversely to the tips of the fingers. The first filling of the bulb and discharging into a spoon is merely to measure the exact capacity of the bulb, and to leave no space for air, though the admixture of a minute quantity of air would not be in the least degree harmful, unless injected into a vein. Practically no air enters, and an objection upon this ground will speedily disappear under a fair test.

The method which I have now employed for a year and a half is, of course crude, and the instrument far from as perfect as it is capable of being made. A well-known instrument maker of Philadelphia is about to manufacture a syringe upon the principle in question. The bulbs will be of two sizes—fifteen and thirty minims. The idea of a screw fitting between the bulb and needle will be abandoned as unnecessary. The needle will be made with a rounded extremity precisely like the glass tubular portion of the medicine dropper, and over this the open extremity of the bulb will fit tightly. A small glass, or porcelain dish, or cup, will be a part of the outfit of exactly the same capacity as the bulb, so that the solution can be made without any preliminary measuring. The bulbs will be made strongly, and of the best quality of rubber. They should fill in ten seconds or less. With the injector carried in my buggy-case I can readily be prepared to give an injection within the short space of two minutes. This includes the fastening of the needle and the twice filling of the bulb. Surely this is sufficiently speedy, but by the help of the instrument maker the time will be greatly lessened. For my part, even if the instrument manufacturers fail to perfect the idea, I have parted company with the ordinary hypodermic syringe. Mine cost fifty-five cents each and give every satisfaction. Others cost from two to three dollars each and are soon out of order. My experience can be made that of any practitioner who will take an interest in the matter, and who will not allow his own lack of skill in its use to lead him into condemnation of the method.

Now, to sum up; the following are the claims made for the crude apparatus in use by myself: 1. It is simpler in construction than the ordinary syringe; hence, more reliable as it never gets out of order. There need be no leakage. It works as well after one or two months of non-use as if used daily. 2. Its cheapness; the cost being about one-third that of the usual instrument, when provided with two needles. 3. Its effectiveness; it accomplishes all that any other contrivance designed for the same purpose will accomplish, with the single exception of being used as a means of injecting ether. Contact with the latter soon destroys india rubber. 4. It affords a means of rendering the delivery of a hypodermic injection in an aseptic manner. The bulbs are very easily cleansed, and easily kept clean. The likelihood of the concurrence of abscess from any thing introduced from without is reduced to a minimum.

